# The Hidden Majority? Exploring the Neurodiversity in CAMHS Eating Disorder Caseloads

**DOI:** 10.1192/bjo.2025.10479

**Published:** 2025-06-20

**Authors:** Rachel Lewis, Megan Davies-Kabir

**Affiliations:** Aneurin Bevan University Health Board, Caerleon, United Kingdom

## Abstract

**Aims:** Emerging research indicates a higher prevalence of Autism Spectrum Disorder (ASD) and Attention-Deficit/Hyperactivity Disorder (ADHD) among individuals with eating disorders (EDs) compared with the general population. Understanding this overlap is crucial for service planning, as neurodiversity assessments and tailored interventions require additional clinical resources. This study aimed to quantify the incidence of neurodiversity within a community specialist Child and Adolescent Mental Health Services (CAMHS) ED caseload to better predict workload demands and inform clinician training and treatment adaptations.

**Methods:** A cross-sectional survey was conducted among clinicians in a specialist CAMHS ED service. Clinicians were asked to report the number of patients on their current caseload with a formal diagnosis of ASD and/or ADHD, as well as those identified as requiring further assessment for these conditions. This methodology provided a snapshot of the prevalence of neurodiversity within active caseloads at the time of data collection.

**Results:** The total caseload was 96 patients. Of these, 22 (23%) had a confirmed diagnosis of ASD and/or ADHD, while 40 (42%) were identified as needing an assessment for neurodevelopmental conditions. In total, 65% of the caseload had either a diagnosis or a suspected diagnosis of ASD, ADHD, or both.

**Conclusion:** Our findings highlight a substantial overlap between eating disorders and neurodiversity in a community specialist CAMHS setting. The high proportion of young people requiring assessment underscores the need for integrated neurodevelopmental screening within ED services. Additionally, these results emphasize the importance of upskilling clinicians in neurodiversity-informed care and adapting treatment models to meet the needs of this population. Addressing these factors will be essential for optimizing clinical outcomes and resource allocation within specialist ED services.

